# Hybridization of Acoustic and Visual Features of Polish Sibilants Produced by Children for Computer Speech Diagnosis

**DOI:** 10.3390/s24165360

**Published:** 2024-08-19

**Authors:** Agata Sage, Zuzanna Miodońska, Michał Kręcichwost, Paweł Badura

**Affiliations:** Faculty of Biomedical Engineering, Silesian University of Technology, Roosevelta 40, 41-800 Zabrze, Poland; zuzanna.miodonska@polsl.pl (Z.M.); michal.krecichwost@polsl.pl (M.K.); pawel.badura@polsl.pl (P.B.)

**Keywords:** computer-assisted speech diagnosis, visual–audio features, sibilants, speech disorders, child speech, hybridization

## Abstract

Speech disorders are significant barriers to the balanced development of a child. Many children in Poland are affected by lisps (sigmatism)—the incorrect articulation of sibilants. Since speech therapy diagnostics is complex and multifaceted, developing computer-assisted methods is crucial. This paper presents the results of assessing the usefulness of hybrid feature vectors extracted based on multimodal (video and audio) data for the place of articulation assessment in sibilants /s/ and /ʂ/. We used acoustic features and, new in this field, visual parameters describing selected articulators’ texture and shape. Analysis using statistical tests indicated the differences between various sibilant realizations in the context of the articulation pattern assessment using hybrid feature vectors. In sound /s/, 35 variables differentiated dental and interdental pronunciation, and 24 were visual (textural and shape). For sibilant /ʂ/, we found 49 statistically significant variables whose distributions differed between speaker groups (alveolar, dental, and postalveolar articulation), and the dominant feature type was noise-band acoustic. Our study suggests hybridizing the acoustic description with video processing provides richer diagnostic information.

## 1. Introduction

Speech disorders are significant barriers to the balanced development of a child. They cause difficulties in learning to read and write and become a source of social withdrawal. Neglecting speech defects that appear in childhood may further deepen them and, as a result, affect adult life. Studies conducted in the 1980s among Polish children reported the occurrence of disorders in approximately 20–30% of six-year-olds [1], while at the beginning of the second decade of the 21st century, this number was estimated at 48% [1,2]. Among speech pathologies, specialists talk about the predominance of one of its types—dyslalia. These are deviations from the norm in the articulation of sounds. The most common type of dyslalia among children is lisping (sigmatism). Sigmatism is the incorrect articulation of dental sounds (sibilants). In Polish, there are 12 sibilants, denoted using the International Phonetic Alphabet (IPA) as /s/, /z/, /ʦ/, /dz/, /ʂ/, /ʐ/, /tʂ/, /dʐ/, /ɕ/, /ʑ/, /ʨ/, /ʥ/. However, in this study, we analyze only two sibilants: /s/ and /ʂ/, regarding their place of articulation.

### 1.1. Background and State of the Art

**Figure 1:**
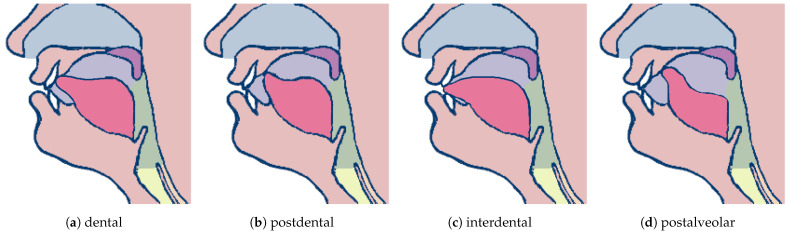
Illustration of various places of articulation (prepared based on [6]).

Speech diagnosis is a complex process. Specialists assess not only free speech (incl. vocabulary, correctness of sentence construction, fluency, and prosody) but also selected anatomical and physiological aspects [2,3]. Features related to motor skills and the structure of articulators (i.e., organs involved in speech generation) include, for example, the degree of shortening of the lingual frenulum, teeth condition, bite, temporomandibular joint efficiency, and the structure of the palate and nasal cavity. Characteristics related to the subject’s physiology include assessment of phonemic and physical hearing and breathing and swallowing functions. The last group describes features associated with the production of individual sounds, mainly regarding the manner and places of articulation and the position of articulators. The place of articulation we analyze in this study is crucial in terms of sibilant formation [2,3]. Not only do the sounds differ in their place of articulation, but slight deviations from the regular position of the articulators may be identified as sigmatism [4]. The literature distinguishes multiple pronunciation patterns (Figure 1), incl. dental articulation (tip of the tongue touches the upper front teeth), interdental articulation (tongue is between upper and bottom teeth), alveolar articulation (tongue apex contacts the alveolar ridge), labiodental articulation (bottom lip raises towards the upper front teeth), or postalveolar articulation (the tip or blade of the tongue approaches or touches the back of the alveolar ridge) [5]. Thus, the multi-layered nature of the diagnosis and its reliance on observation of the articulators’ functioning requires a specialist’s services and can be time-consuming. A properly selected therapeutic path increases the effectiveness of treatment and shortens its duration. Therefore, developing computer methods supporting speech therapy diagnostics is crucial for many reasons mentioned above.

Computer pronunciation analysis is a broad issue regarding the purpose of such solutions, the data, and the methods used. Most solutions to date have focused on analyzing normative pronunciation, including learning foreign languages, speech recognition, and speaker identification, as well as recognizing and classifying individual sounds. The recent AI-driven models for automated speech recognition (ASR) involve audio and video data and are mostly trained on adult speech, reaching considerable performance [7,8,9,10]. Solutions strictly supporting diagnostics and speech therapy form a much narrower subset. Some available concepts feature high spatial and temporal accuracy yet are invasive and require significant experimental resources or costs. They include, among others, electromagnetic articulography [11,12], used to observe articulators in an alternating magnetic field, or electropalatography [13], which monitors the tongue–palate contact during pronunciation. Both are not entirely contactless and interfere with the oral cavity of the subject. Many researchers use the acoustic signal recorded with one or more microphones in various configurations [14,15,16,17]. The literature on linguistics and phonetics offers much information on the acoustics of sibilant sounds. Based on this knowledge, researchers have analyzed the possibilities of using the acoustic signal, e.g., in automatic recognition of sounds (although mainly in normal pronunciation so far). Numerous studies focus on searching for acoustic parameters that distinguish individual fricative sibilants [18,19,20,21]. Due to the specific nature of sibilants, research usually concerns a limited subset of sounds occurring in a given language. Moreover, a relatively small number of works describing acoustic analysis concern child speech [15,21,22,23,24,25].

The analysis of dental sounds in the literature often employs the processing of the signal spectrum. The use of spectral moments appears in many works [19,20]. Researchers have reported that the center of gravity of the spectrum shifts in sibilants depending on the place of articulation [18]. Another group of acoustic parameters describing dental sounds are features related to frication noise. Some works focused on searching for differences between sounds in the frequencies and amplitudes of noise formants that appear in the spectrum above 2 or 3 kHz [22,26,27]. Others used width and lower limit of the noise band, energy differences in individual frequency bands, frication duration or cepstral coefficients in the noise band, and noise formant ratios [22,26,27].

According to our knowledge, no studies have used the potential of image data to represent child pronunciation. Some pathological patterns related to the motion or positioning of organs may be visible in video recordings. There are aids in speech therapy that show similarities to this approach. Specialists use sets of photographs (or drawings) presenting subsequent stages of pronouncing individual sounds, called labiograms [2]. The boards help practice the correct arrangement of organs. Using such materials suggests the usefulness of building computer methods based on this modality. However, developing computerized solutions often requires finding numerical features describing various aspects of objects. It might be reasonable to extract parameters similar to radiomic features based on pictures presenting articulators. Radiomics uses the extraction of quantitative parameters from medical images. The literature divides radiomic features into statistical (including those dependent on histogram and texture), mathematical-model-based, spectral, and shape parameters [28].

### 1.2. Contribution of the Paper

In this paper, we propose hybridizing acoustic and visual features to assess articulation. Such combining may indicate patterns in various aspects (e.g., not seen in one modality but supplemented by the other) and expand diagnostic information. Changes in the place of articulation often yield an abnormal realization of sounds. We analyzed the articulation of two fricative Polish sibilants: /s/ and /ʂ/ produced by children aged 5–8 (183 and 178 speakers, respectively). Apart from employing well-known features for sibilant analysis, like parameters based on the entire band (MFCCs, spectral moments) or noise band (cepstral coefficients, fricative formants), we proposed a set of visual features describing the texture and shape of selected articulators. We calculated image parameters based on the visual segmentation of lips, mouth, and tongue reported in [29]. To our knowledge, none of the solutions described in the literature reported a similar approach. Finally, the results of the statistical analysis, including the Mann–Whitney U test and the Kruskal–Wallis test, followed by post hoc analysis, provided the basis for assessing the potential of our hybrid concept.

### 1.3. Structure of the Paper

The remainder of the paper is structured as follows: Section 2 describes the materials and methods, covering the data recording protocol, speech corpus structure, image and acoustic data preprocessing workflow, feature extraction, and statistical analysis procedures. Section 3 presents the results of the Mann–Whitney U and the Kruskal–Wallis tests, followed by post hoc analysis. Section 4 discusses our results, and Section 5 concludes the paper.

## 2. Materials and Methods

### 2.1. Materials

**Figure 2:**
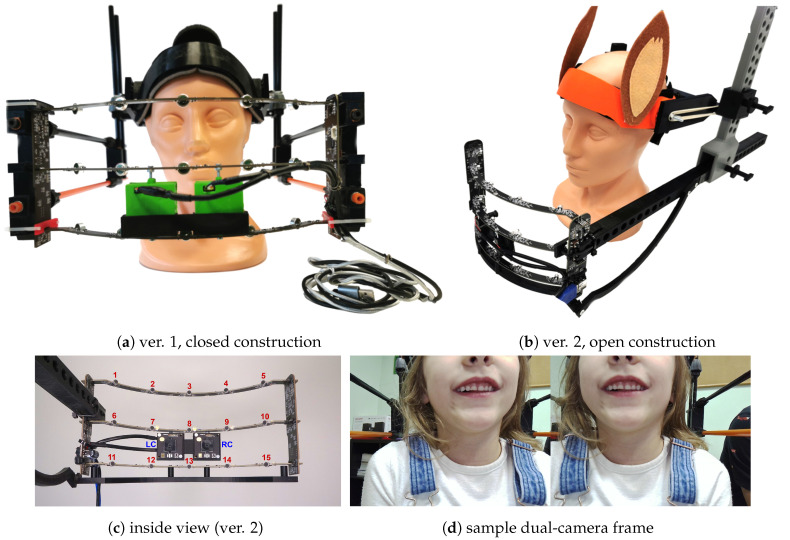
Multimodal data acquisition device (MDAD) construction and operation [30]: (**a**) closed construction [32]; (**b**) open construction, recent version; (**c**) inside view to the measuring part; red numbers represent the microphone (audio channel) numbers, “LC” and “RC” indicate the left and right camera; (**d**) sample dual-camera view.

We collected a multimodal database of child pronunciation in cooperation with speech therapy specialists [30]. Our team performed speech therapy examinations and data recording sessions in six kindergarten and school facilities. The examination had three stages [31]: (1) registering the child’s speech while naming pictures visible on the screen with a dedicated multimodal data acquisition device (MDAD, Figure 2) [30,32]; (2) recording the speaker while repeating given words and logatomes following the speech therapist and during a set of speech therapy exercises (incl. tongue movements, smiling, swallowing); (3) speech therapy examination performed by a speech–language pathologist (SLP) according to the dedicated diagnostic protocol for sigmatism-related pronunciation assessment (no recording in this stage).

The multimodal recording device was designed for the project [30,32]. It records the audio signal from a semicylindrical microphone array (15 channels, spatially distributed) and captures the video of the lower part of the speaker’s face using a double-camera module. We started with a closed mask construction (Figure 2a). The recent version of the tool has a lighter open construction (Figure 2b). The recording session began with the device placed safely and comfortably on the child’s head. The speech corpus included Polish sibilant-related material consisting of 51 words and 12 logatomes, including all 12 Polish sibilant sounds in various configurations, environments, and word positions. As a result, we collected an extensive multimodal database including 201 children aged 4–8, along with the corresponding speech therapy diagnoses from two independent experts [30]. However, our speech corpus was narrower in this study as we focused on two selected sibilants (/s/, /ʂ/). It included seven words, one logatome containing /s/ and 12 words and a single logatome with /ʂ/ (Table 1).

### 2.2. Methods

**Figure 3:**
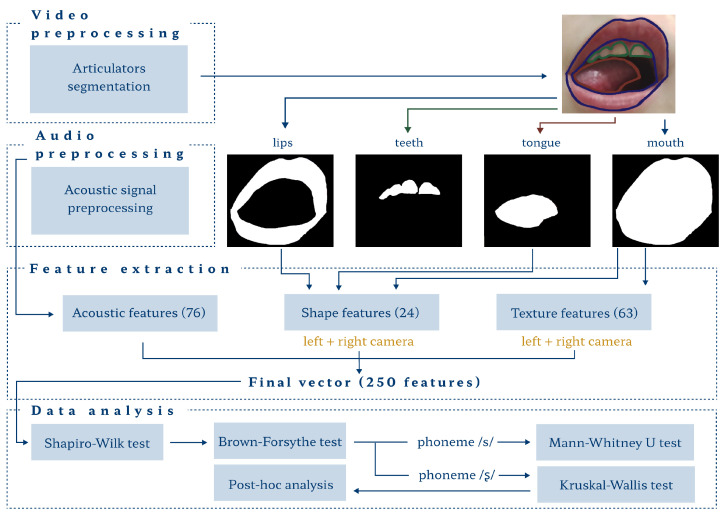
Schematic overview of the workflow.

Our workflow included data preprocessing, feature extraction, and statistical analysis (Figure 3). In the first two stages, we proposed separate paths for image and audio data. In the last stage, the methods employed combined (audio–visual) vectors.

#### 2.2.1. Data Preprocessing

**Figure 4:**
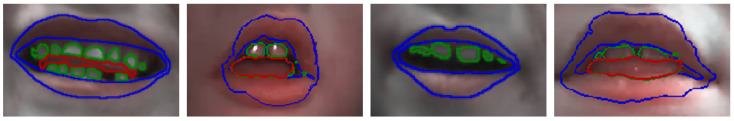
Examples of segmentation results for sample frames during sibilant articulation. The lips are marked in blue, the teeth in green, and the tongue in red. The teeth region was not used in this work.

Before preprocessing, we synchronized the visual and acoustic data in time. Thus, the methods operated on video frames and acoustic data representing the exact sibilant-articulation segment. Before data processing, an expert indicated the beginning and end of the sibilant articulation period in the audio files (acoustic segmentation process). We performed a two-stage visual segmentation to delineate lips, mouth, tongue, and teeth: (1) object detection using YOLOv6 (you only look once) to crop images to bounding-box covering mouth area, and (2) segmentation with DeepLabv3+ model on mouth-restricted frames [29,33,34]. We reviewed and rejected low-quality delineations (incl. segmentations leaked on other objects and insufficiently segmented organs). This procedure strengthened the credibility of the method and statistical analysis. The sample segmentation results are given in Figure 4.

The second path preprocessed the acoustic signal of the corresponding segments. This study uses a single-channel signal processing approach (the central microphone, #8 in Figure 2c). We started with data normalization within the sibilant segment to obtain values in the 0–1 range according to the following equation:

(1) $$x\left(n\right)=\frac{x_{o}\left(n\right)}{x_{max}-x_{min}},$$

where $x_{o}\left(n\right)$ is the input, and $x_{max},x_{min}$ are the maximum and minimum samples in the given segment, respectively. The normalized signal was partitioned into 33-ms frames with no overlap and windowed with a Hamming window. To hybridize multimodal features, we set the frame duration to match the video frame rate. The sampling frequency was 44.1 kHz.

#### 2.2.2. Feature Extraction

In this study, we proposed using audio and image features to search for potential differences in their distribution between various articulation patterns in /s/ and /ʂ/. We extracted 87 visual features for a single view based on the delineations of the articulators. It included two-dimensional textural parameters of the mouth region of interest (ROI) and features related to lips, mouth, and tongue geometry. Our segmentation method also indicated the area of teeth. However, based on the suggestions of our SLPs, we discarded the teeth from further analysis as potentially problematic in preliminary studies. Missing teeth are a developmental standard and do not necessarily indicate speech problems. The textural parameters included intensity-related and histogram-related global features, gray level co-occurrence matrix (GLCM) features, gray level size zone (GLSZM) features, gray level run length matrix (GLRLM) features, and neighboring gray-tone difference matrix (NGTDM) features. The number of gray levels was 32, as the aim was to find general patterns. We gathered all the image parameters in Table 2. As mentioned, our recording tool had two cameras, so all visual parameters appeared twice for the left and right cameras (i.e., 174 variables in total).

Simultaneously, we extracted parameters from the audio signal. The feature extraction involved three types of acoustic cues: time-domain features (4), full-band spectral acoustic features (24), and noise-band spectral acoustic features (48). Table 3 presents all the acoustic parameters employed in this study.

**Figure 5:**
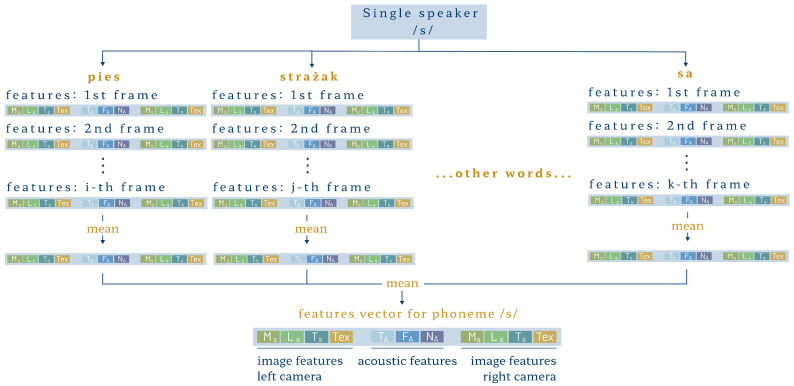
Feature aggregation for a single speaker and one sibilant. $M_{S}$ —mouth shape features, $L_{S}$ —lips shape features, $T_{S}$ —tongue shape features, Tex —texture features of mouth area, $T_{A}$ —time-domain acoustic features, $F_{A}$ —full-band spectral acoustic features, $N_{A}$ —noise-band spectral acoustic features.

Video and audio segments embracing articulated dental sounds consisted of few frames, but their length varied between speakers. We calculated vectors of audio–visual features for each frame. Therefore, a single sound produced a matrix of parameters, and each speaker made a given sound several times. According to the procedure presented in Figure 5, the parameters for individual sibilants resulted from averaging within each speaker. This way, we obtained one feature vector for each speaker to ensure data independence in statistical analysis. Finally, the individual vectors were reduced by cropping 25% of all frames at the beginning and 25% at the end to remove possible occurrences of preceding or following sounds, silence, or background noise.

#### 2.2.3. Statistical Analysis

The analysis consisted of two stages. First, we performed data mining to determine the distributions of the variables. Then, we verified the hypotheses using statistical tests. The significance level α was 0.05 in all experiments. In addition to the statistical significance, we provided the effect size to measure the magnitude of differences between group means or medians. We assumed the following interpretation of the effect size [56,57]:

- The biserial correlation coefficient *rb* for the Mann–Whitney U test: low—below 0.39, medium—0.40–0.59, high—above 0.60 (absolute value).
-
$\eta^{2}$ for the Kruskal–Wallis test: low—0.01–0.05, medium—0.06–0.13, high—above 0.14.

This study focused on the sounds /s/ and /ʂ/ regarding place of articulation analysis. During the examinations in preschool facilities, we recorded the speech of 200 children. SLPs observed six various patterns of articulation for sibilant /s/ (dental, alveolar, interdental, addental, labiodental, other) and seven for /ʂ/ (dental, postalveolar, alveolar, interdental, labiodental, addental, and other). However, we rejected observations with insufficiently accurate visual segmentation. Thus, sound /s/ was produced by 183 speakers, and /ʂ/ by 178. Before statistical analysis, we also assessed disparities between the number of observations in different articulation patterns. After eliminating small groups, further steps addressed two types of articulation in /s/ (dental and interdental) and three in /ʂ/ (dental, alveolar, and postalveolar). The data summary is given in Table 4.

For each considered variable, the Shapiro–Wilk (SW) test [58] was performed to determine the normality of distribution. We tested all variables in both sounds and all articulation patterns. In most cases, the SW test required the rejection of the null hypothesis of normality of distribution. In the case of features with high skewness, further analysis used the logarithm of their values. Due to the dominance of asymmetric distributions, we considered only non-parametric tests in the following steps. Therefore, to analyze the homogeneity of variance, we used a non-parametric Brown–Forsythe test [59]. Although *p* values above 0.05 prevailed, which provided the basis for accepting the null hypothesis, the result suggested heterogeneity of this measure in some features. For those variables, we calculated the ratio of variances between each group of observations to indicate the diversity of scales. A variance ratio above 10.0 or less than 0.1 excluded the feature from further analysis. The same was true for multigroup analyses, even if the rule was broken only between one pair.

The final stage included a set of statistical tests to assess the discrimination capability of individual features. Due to the predominance of asymmetric distributions, we did not use the analysis of means. Instead, we employed a non-parametric analysis to assess the equality of medians. In the binary case, it was the Mann–Whitney U test (U MW) [58,60], and in multi-class problems, the Kruskal–Wallis test (KW) [58,61]. Since the KW test only provides information that at least one tested group is different from another, we performed the post hoc Bonferroni test to determine which groups differ [62].

## 3. Results

The presentation of the results includes general findings concerning differences between pronunciation patterns indicated by the assessment of median equality. As mentioned, most variables had asymmetric distributions. Therefore, the analysis employed non-parametric tests. We used the Mann–Whitney U test for sibilant /s/ to search for inter-class differences (dental and interdental articulation) in visual and acoustic features. In sound /ʂ/ with three realizations (alveolar, dental, postalveolar), the analysis employed the Kruskal–Wallis test. We discuss further only the parameters with a *p*-value below 0.05 (statistically significant differences in feature distributions between given articulation patterns).

**Figure 6:**
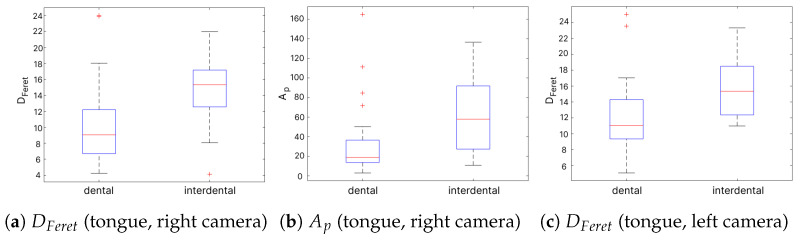
Box plots for two features with the highest effect size in sibilant /s/: tongue’s $D_{Feret}$ and $A_{p}$ .

Thirty-four features proved to significantly differentiate dental and interdental pronunciation patterns in the /s/ sound (Table 5). Among them, 24 were image-based, 7 of which concerned the shape of the tongue, and 17 were related to the texture of the mouth. Ten parameters considered acoustics of frication noise. Six visual features appeared for both the left and right cameras. However, the largest effect size (medium level, according to the approach presented in Section 2.2.3) was obtained in visual features describing the tongue shape. The distribution of features proved the differences between pronunciation patterns (see Figure 6). Medians of three visual features with the highest size effect (tongue’s $D_{Feret}$ from the left and right camera and $A_{p}$ from the right camera) observably distinguished dental and interdental speech. Interdental articulation showed higher medians in all cases. It was likely related to the more frequent occurrence of a tongue and its larger area.

**Figure 7:**
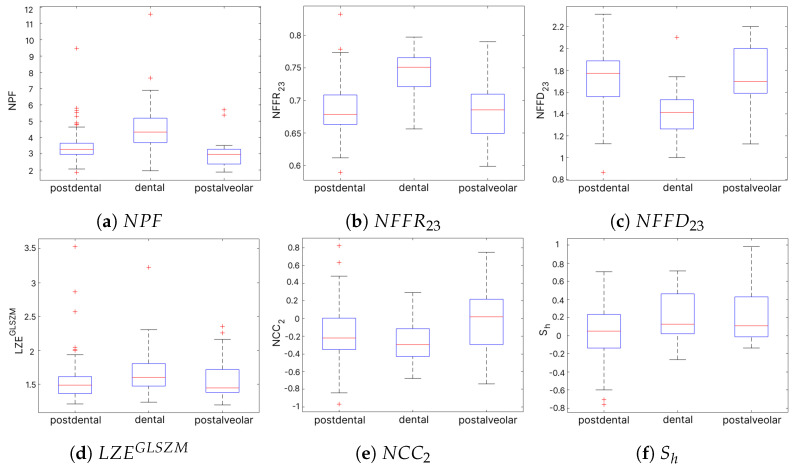
Box plots for selected statistically significant features in sibilant /ʂ/: (**a**–**c**) present distributions of three variables with the highest effect size $\eta^{2}$ , and (**d**–**f**) with the lowest.

In the second experiment, 49 variables significantly differentiated at least one pronunciation pattern in sibilant /ʂ/ (Table 6). Forty-three were acoustic (27 noise-band, 14 full-band, and 2 time-domain) and six described image texture. The Bonferroni test indicated 43 variables showing differences between at least one pair of groups (1–2: alveolar–dental, 1–3: alveolar–postalveolar, 2–3: dental–postalveolar). Nine features varied between all articulation patterns. However, we found most differences between alveolar vs. postalveolar (35 features) and dental vs. postalveolar pronunciation (33 variables). The alveolar vs. dental pair indicated 15 parameters. Figure 7 presents the distribution of three variables with the highest effect size and three with the lowest. We observed that medians of NPF , $NFFR_{23}$ , and $NNFD_{23}$ (Figure 7a–c) were noticeably different between at least one pair of articulation patterns. Considering the parameters of smaller effect size (Figure 7d–f), the dissimilarities are relatively subtle.

## 4. Discussion

For each speaker and sibilant, we extracted 87 visual parameters and 76 acoustic features. According to the state-of-the-art and literature review, we found several studies regarding contact (e.g., electropalatography) and non-contact (e.g., audio signal) data registering protocols for sibilant articulation analysis. None of them, however, used image data. According to the idea of labiograms, some pronunciation patterns should be visible in the motion and placement of speech organs, and their analysis can contribute to the diagnostic process. Thus, in previous studies, we proposed a segmentation tool to extract lips, tongue, and mouth (lips and the area in between). In this study, we employed automated delineations and investigated hybrid visual–acoustic features for CASD purposes.

We expected an incorrect motor pattern to be most noticeable in lips and mouth movement, also resulting from disordered activity of other organs, e.g., the tongue or jaw. Both are constantly visible in video recordings and easy to segment, which is not the case with the tongue, often hidden behind lips or teeth. Our study shows that greater visibility of the tongue or its unusual positioning may be related to incorrect pronunciation. This observation is valid when assessing the place of articulation in sound /s/, where we examined the differences between dental and interdental realization. The highest size effect was indicated in features describing the tongue shape. The tongue object featured increased area, diameters, or axes in interdental productions compared to dental. Texture features are another large group significantly differentiating dental and interdental articulation in /s/, although with relatively small effect sizes. We calculated the texture metrics using 32 gray levels. The idea behind such a selection was to search for general, coarse textural relations. Inter-speaker differences and external conditions (mainly lighting) could decrease the repeatability of patterns distinguishing articulation. The Mann–Whitney U test results also indicated 11 audio features (all noise-band related), lower in the effect size than in tongue-shape features but mostly higher than in texture parameters. Considering /s/ analysis only, the hybridization of visual and audio features is valuable as both appear statistically significant, with the predominance of the former. Finding differences between dental and interdental articulation is essential, as the latter is not a developmental norm in Polish, and its early detection can make the therapy more efficient.

On the other hand, the analysis of sibilant /ʂ/ showed the dominance of acoustic features among all that were statistically significant. Only six were visual (textural) and had a relatively small effect size. The post hoc analysis indicated most differences between the alveolar and dental and between postalveolar and dental articulation. Substantial representation of noise-band features might result from shifts of the noise band in each articulation pattern. In this experiment, we did not include the interdental realization of /ʂ/ possibly higher and more frequent tongue appearance. The articulations considered in the assessment of /ʂ/ place of articulation (alveolar, dental, and postalveolar) embraced the contact of the frontal part of the tongue with the upper teeth or gums. Thus, the tongue shape features might not have been efficient in distinguishing pronunciation patterns. We expected that distortions in the motion pattern should be reflected in lips or mouth shape features. However, our analysis did not prove that assumption.

Even though the analysis proved that adding image-based parameters broadens diagnostic information, the results also indicate that combining visual and acoustic features is beneficial only in selected sibilants and articulations. While both types of features appear in sound /s/, the acoustic ones dominate in /ʂ/. The ways of articulating these sounds differ, so the differences may concern different aspects, e.g., incorrect positioning of the lips or tongue. They may also be imperceptible in video recordings yet noticeable in acoustics.

The extensive research conducted in several preschool institutions showed that distorted production of sibilants is frequent, of various intensities, and often results from different causes. Considering the scale of the problem and the fact that children at this stage can do much work supervised by a specialist, the development of CASD methods is necessary. The preliminary results presented in this paper indicate the potential of hybridization of visual and audio features in searching for differences in the place of articulation between various realizations of sibilants. That concept benefits in richer diagnostic information. Nevertheless, this study had some limitations, and the proposed idea still has many possibilities for development. We want to extend our research by adding other sounds and articulation features. This preliminary study is a good starting point for constructing expert systems supporting the speech therapy diagnosis of sigmatism. Finding the most relevant parameters opens perspectives for developing classification tools for CASD. This work focused on sibilants. However, the audio–visual approach may also be the basis for analyzing pronunciation in other groups of Polish sounds. The dependence on the segmentation and aggregation procedures preceding statistical analysis remains challenging in such a study, as possible outliers might impact the aggregation outcomes. Regardless of the development direction, expanding the available solutions with further tests is valuable for improving speech therapy diagnosis and therapy.

## 5. Conclusions

In this paper, we addressed combining acoustic and visual features to analyze the place of articulation in Polish sibilants /s/ and /ʂ/. The results justify searching for relevant features in different representations of articulation. The Mann–Whitney U tests indicated variables (both visual and acoustic) that significantly differentiate dental and interdental articulation patterns in /s/. The predominating parameters were visual, including tongue shape and mouth texture features. The Kruskal–Wallis test also showed statistically significant differences between alveolar, dental, and postalveolar pronunciations in /ʂ/, yet with the predominance of acoustic noise-band features.

## Figures and Tables

**Table 1:** Set of words containing /s/ and /ʂ/ used in the study.

Word (PL)	IPA	Sibilant	Word (EN)	Word (PL)	IPA	Sibilant	Word (EN)
pie**s**	/p^j^εs/	/s/	dog	**sz**afa	/’ʂafa/	/ʂ/	wardrobe
**s**trażak	/’straʐak/	/s/	firefighter	**sz**ufelka	/ʂu’fεlka/	/ʂ/	dustpan
**s**amolot	/sa’mɔlɔt/	/s/	airplane	nó**ż**	/’nuʂ/	/ʂ/	knife
**s**ałata	/sa’wata/	/s/	lettuce	wą**ż**	/’vɔ $\tilde{w}$ ʂ/	/ʂ/	snake
para**s**ol	/pa’rasɔl/	/s/	umbrella	ksią**ż**ka	/’kɕɔ $\tilde{w}$ ʂka/	/ʂ/	book
la**s**	/’las/	/s/	forest	leka**rz**	/’lεkaʂ/	/ʂ/	physician
cia**s**tka	/’ʨastka/	/s/	cookies	**sz**nurek	/’ʂnurεk/	/ʂ/	cord
**s**adzawka	/sa’dzafka/	/s/	pond	kucha**rz**	/’kuxaʂ/	/ʂ/	cook
sa	/sa/	/s/	—	**sz**alik	/’ʂalik/	/ʂ/	scarf
				ka**sz**a	/’kaʂa/	/ʂ/	groats
				ko**sz**yk	/’kɔʂɪk/	/ʂ/	basket
				kalo**sz**e	/ka’lɔʂε/	/ʂ/	rain boots
				**sz**a	/ʂa/	/ʂ/	—

**Table 2:** Visual features used in the study: GLE stands here for gray level emphasis, and GLI means gray level intensity.

Textural Features					
Global: histogram [35,36,37,38]		Global: intensity [35,36,37,38]		GLCM [36,39,40,41]	
$E_{h}$	Energy	$I_{E}$	Energy	$ASM^{GLCM}$	Angular second moment
$H_{h}$	Entropy	$I_{\sigma^{2}}$	Variance	$Con^{GLCM}$	Contrast
$\sigma_{h}^{2}$	Variance	$I_{\sigma }$	Standard deviation	$Ent^{GLCM}$	Entropy
$\sigma_{h}$	Standard deviation	$I_{min}$	Minimum GLI	$Mean^{GLCM}$	Mean
$S_{h}$	Skewness	$I_{max}$	Maximum GLI	$Var^{GLCM}$	Variance
$K_{h}$	Kurtosis	$I_{\mu }$	Mean GLI	$Cor^{GLCM}$	Correlation
		$I_{med}$	Median GLI	$Hom^{GLCM}$	Homogeneity
		$I_{R}$	Range of GLI	$Dis^{GLCM}$	Dissimilarity
		$I_{MAD}$	Mean absolute deviation	$AC^{GLCM}$	Autocorrelation
		$I_{rMAD}$	Robust MAD	$SA^{GLCM}$	Sum average
		$I_{RMS}$	Root mean square		
		$I_{p10}$	10th percentile of GLI		
		$I_{p90}$	90th percentile of GLI		
		$I_{IQR}$	Interquartile range		
		$I_{S}$	Skewness		
		$I_{K}$	Kurtosis		
GLRLM [28,41,42,43]		GLSZM [41,43,44]		NGTDM [41,45]	
$SRE^{GLRLM}$	Short run emphasis	$SZE^{GLSZM}$	Short zone emphasis	$Coar^{NGTDM}$	Coarseness
$LRE^{GLRLM}$	Long run emphasis	$LZE^{GLSZM}$	Large zone emphasis	$Con^{NGTDM}$	Contrast
$GLN^{GLRLM}$	Gray-level non-uniformity	$GLN^{GLSZM}$	Gray-level uniformity	$Bus^{NGTDM}$	Busyness
$RLN^{GLRLM}$	Run length non-uniformity	$ZSN^{GLSZM}$	Zone size non-uniformity	$Com^{NGTDM}$	Complexity
$RP^{GLRLM}$	Run percentage	$ZP^{GLSZM}$	Zone percentage	$TS^{NGTDM}$	Texture strength
$LGLRE^{GLRLM}$	Low gray-level run emphasis	$LGZE^{GLSZM}$	Low gray-level zone emphasis		
$HGRE^{GLRLM}$	High gray-level run emphasis	$HGZE^{GLSZM}$	High gray-level zone emphasis		
$SRLGLE^{GLRLM}$	Short run low GLE	$SZLGE^{GLSZM}$	Small zone low GLE		
$SRHGLE^{GLRLM}$	Short run high GLE	$SZHGE^{GLSZM}$	Small zone high GLE		
$LRLGLRE^{GLRLM}$	Long run low GLE	$LZLGE^{GLSZM}$	Large zone low GLE		
$LRHGLRE^{GLRLM}$	Long run high GLE	$LZHGE^{GLSZM}$	Large zone high GLE		
$GLV^{GLRLM}$	Gray-level variance	$GLV^{GLSZM}$	Gray level variance		
$RV^{GLRLM}$	Run variance	$RLV^{GLSZM}$	Zone size variance		
**Shape features [38,41,46,47]**					
		$A_{p}$	Pixel surface		
		*P*	Perimeter		
		*S*	Sphericity		
		SD	Spherical disproportion		
		$Ax_{major}$	Major axis length		
		$Ax_{minor}$	Minor axis length		
		*E*	Elongation		
		$D_{Feret}$	Maximum Feret diameter		

**Table 3:** Acoustic features used in the study.

Acoustic Features					
Time domain [48,49,50]		Spectral: full band [51,52,53]		Spectral: noise band [22,54,55]	
$ZCR_{t}$	Zero-cross rate	$SCen_{f}$	Spectral centroid	$NFF_{1–4}$	Fricative formant frequencies
$STE_{t}$	Short-term energy	$SSpr_{f}$	Spectral spread	$NFFL_{1–4}$	Fricative formant levels
$P_{t}$	Pitch	$SSk_{f}$	Spectral skewness	$NFFR_{12}$ , …, $NFFR_{34}$	Fricative formant frequency ratio
$HR_{t}$	Harmonic ratio	$SCr_{f}$	Spectral crest	$NFLR_{12}$ , …, $NFLR_{34}$	Fricative formant level ratio
		$SD_{f}$	Spectral decrease	$NCC_{0–12}$	Noise cepstral coefficients
		$SE_{f}$	Spectral entropy	$NE_{0–9}$	Noise energy
		$SFla_{f}$	Spectral flatness	$NFFD_{12,23,34}$	Fricative formant distances
		$SFlx_{f}$	Spectral flux	NPA	Peak amplitude
		$SRP_{f}$	Spectral rolloff-point	NPF	Peak frequency
		$SKurt_{f}$	Spectral kurtosis		
		$SSl_{f}$	Spectral slope		
		$MFCC_{0–12}$	MFCC coefficients		

**Table 4:** Description of articulation patterns [5] with the number of observations in each group.

Class	Description	Observations	
		/s/	/ʂ/
dental	tip of the tongue touches the upper front teeth	113	27
alveolar	tongue apex contacts the alveolar ridge	—	106
interdental	tongue is between upper and bottom teeth	31	—
postalveolar	the tip or blade of the tongue approaches or touches the back of the alveolar ridge	—	29

**Table 5:** The results of the Mann–Whitney U test in sibilant /s/ and the point of articulation assessment of dental and interdental pronunciation. V and A in the Data column denote video and audio, respectively. Column Type indicates the category of features.

No.	Feature	Data	Type	Camera	*p*	H	rb	No.	Feature	Data	Type	Camera	*p*	H	rb
1	$D_{Feret}$	V	tongue	right	0.003	611.5	0.439	18	$Con^{GLCM}$	V	texture	left	0.017	7283.0	0.209
2	$A_{p}$	V	tongue	right	0.004	614.5	0.429	19	$NE_{1}$	A	noise		0.013	7680.0	0.208
3	$D_{Feret}$	V	tongue	left	0.003	580.0	0.426	20	$Dis^{GLCM}$	V	texture	right	0.024	7260.0	0.198
4	$Ax_{major}$	V	tongue	right	0.004	616.0	0.424	21	$Coar^{NGTDM}$	V	texture	right	0.026	6473.0	0.195
5	$Ax_{minor}$	V	tongue	left	0.004	583.5	0.416	22	$Coar^{NGTDM}$	V	texture	left	0.027	6475.0	0.194
6	$Ax_{major}$	V	tongue	left	0.014	604.0	0.355	23	$Con^{NGTDM}$	V	texture	right	0.028	7251.0	0.193
7	SD	V	tongue	right	0.017	637.0	0.354	24	$Con^{GLCM}$	V	texture	right	0.030	7246.0	0.191
8	$FFRL_{14}$	A	noise		0.001	8906.0	0.290	25	$Hom^{GLCM}$	V	texture	left	0.030	6483.0	0.190
9	$FFRL_{13}$	A	noise		0.001	8866.0	0.274	26	$ZP^{GLSZM}$	V	texture	left	0.037	7232.0	0.184
10	$NE_{0}$	A	noise		0.004	7598.0	0.242	27	$SZE^{GLSZM}$	V	texture	left	0.038	7230.0	0.183
11	$FFL_{1}$	A	noise		0.005	7617.0	0.234	28	$NE_{9}$	A	noise		0.030	8640.0	0.182
12	$NE_{7}$	A	noise		0.005	8765.0	0.233	29	$ZSN^{GLSZM}$	V	texture	left	0.040	7225.0	0.180
13	$NE_{8}$	A	noise		0.006	8762.0	0.231	30	$GLV^{GLSZM}$	V	texture	left	0.040	7225.0	0.180
14	$FFRL_{12}$	A	noise		0.009	8731.0	0.219	31	$LZE^{GLSZM}$	V	texture	left	0.044	6509.0	0.177
15	$Dis^{GLCM}$	V	texture	left	0.015	7291.0	0.213	32	$Com^{NGTDM}$	V	texture	left	0.048	7212.0	0.174
16	$FFL_{4}$	A	noise		0.012	8709.0	0.210	33	$SZE^{GLSZM}$	V	texture	right	0.048	7212.0	0.174
17	$Con^{NGTDM}$	V	texture	left	0.017	7284.0	0.210	34	$LRHGE^{GLRLM}$	V	texture	left	0.049	7210.0	0.173

**Table 6:** The results of the Kruskal–Wallis test and the Bonferroni post hoc analysis in sibilant /ʂ/ and the articulation pattern assessment in (1) alveolar, (2) dental, and (3) postalveolar pronunciation. V and A in Data column denote video and audio, respectively. Column Type indicates the category of features.

No.	Feature	Data	Type	Camera	*p*	H	$\eta^{2}$	Post Hoc		
								1–2	1–3	2–3
1	NPF	A	noise		<0.001	35.575	0.211	0.339	1.000	0.062
2	$NFFR_{23}$	A	noise		<0.001	34.603	0.205	1.000	0.198	0.044
3	$NFFD_{23}$	A	noise		<0.001	28.196	0.165	0.228	1.000	0.127
4	$NFFRL_{14}$	A	noise		<0.001	27.991	0.163	0.220	1.000	0.124
5	$ZCR_{t}$	A	time-domain		<0.001	27.628	0.161	0.044	<0.001	0.013
6	$NFFRL_{13}$	A	noise		<0.001	27.571	0.161	1.000	<0.001	<0.001
7	$Skurt_{f}$	A	full-band		<0.001	25.331	0.147	0.859	0.004	<0.001
8	$NNE_{1}$	A	noise		<0.001	24.294	0.140	0.064	1.000	0.129
9	$NFFD_{12}$	A	noise		<0.001	23.844	0.137	0.038	0.104	1.000
10	$SFlx_{f}$	A	full-band		<0.001	22.688	0.130	0.047	0.002	0.213
11	$MFFC_{10}$	A	full-band		<0.001	21.496	0.123	0.112	0.004	0.149
12	$NNE_{2}$	A	noise		<0.001	21.445	0.122	0.035	<0.001	0.016
13	$NFFR_{12}$	A	noise		<0.001	21.426	0.122	0.900	0.010	0.027
14	$NFFR_{24}$	A	noise		<0.001	20.940	0.119	0.477	0.003	0.020
15	$NFFL_{3}$	A	noise		<0.001	20.116	0.114	0.542	0.011	0.062
16	$NFFL_{1}$	A	noise		<0.001	19.272	0.109	0.459	0.002	0.012
17	$NFFL_{4}$	A	noise		<0.001	18.764	0.105	0.178	<0.001	0.001
18	$NFF_{2}$	A	noise		<0.001	17.904	0.100	1.000	0.006	<0.001
19	$MFFC_{2}$	A	full-band		<0.001	17.856	0.100	0.038	<0.001	<0.001
20	$SFla_{f}$	A	full-band		<0.001	17.835	0.100	1.000	0.258	0.027
21	$NNE_{0}$	A	noise		<0.001	16.313	0.090	0.014	<0.001	<0.001
22	$MFFC_{11}$	A	full-band		0.001	15.033	0.082	0.104	1.000	0.120
23	$NNE_{6}$	A	noise		0.001	14.523	0.079	1.000	0.002	<0.001
24	$NFFRL_{23}$	A	noise		0.001	14.148	0.076	0.313	<0.001	0.001
25	$NNE_{5}$	A	noise		0.001	14.124	0.076	0.040	<0.001	0.005
26	$NNE_{3}$	A	noise		0.001	13.477	0.072	0.014	<0.001	0.026
27	$MFFC_{8}$	A	full-band		0.002	12.644	0.067	0.346	0.041	0.000
28	$MFFC_{5}$	A	full-band		0.003	11.474	0.060	0.033	1.000	0.102
29	$SSpr_{f}$	A	full-band		0.003	11.410	0.059	1.000	<0.001	<0.001
30	$MFFC_{0}$	A	full-band		0.006	10.350	0.053	0.617	<0.001	<0.001
31	$MFFC_{3}$	A	full-band		0.009	9.464	0.047	0.120	0.011	0.317
32	$NFFR_{14}$	A	noise		0.010	9.127	0.045	0.036	<0.001	<0.001
33	$NNE_{7}$	A	noise		0.011	9.011	0.044	0.012	<0.001	0.001
34	$MFFC_{6}$	A	full-band		0.012	8.835	0.043	1.000	0.007	0.001
35	$NFFRL_{12}$	A	noise		0.014	8.591	0.041	1.000	0.031	0.041
36	$NCC_{0}$	A	noise		0.017	8.100	0.038	0.619	0.010	0.000
37	$SRP_{f}$	A	full-band		0.020	7.789	0.036	1.000	<0.001	<0.001
38	$NFFRL_{24}$	A	noise		0.021	7.732	0.036	0.024	<0.001	0.047
39	$LRHGE^{GLRLM}$	V	texture	left	0.036	6.663	0.033	1.000	<0.001	<0.001
40	$LRHGE^{GLRLM}$	V	texture	right	0.036	6.662	0.033	1.000	0.004	<0.001
41	$NFF_{1}$	A	noise		0.028	7.182	0.033	0.147	0.654	0.002
42	$GLV^{GLSZM}$	V	texture	right	0.040	6.423	0.031	0.091	0.001	0.032
43	$STE_{t}$	A	time-domain		0.033	6.848	0.030	0.025	<0.001	0.097
44	$NNE_{9}$	A	noise		0.033	6.795	0.030	0.041	0.012	0.735
45	$SSl_{f}$	A	full-band		0.034	6.761	0.030	0.034	0.126	1.000
46	$I_{S}$	V	texture	left	0.046	6.163	0.029	0.591	0.683	0.018
47	$LZE^{GLSZM}$	V	texture	left	0.047	6.107	0.029	0.111	0.041	0.915
48	$NCC_{2}$	A	noise		0.037	6.587	0.029	0.420	1.000	0.054
49	$S_{h}$	V	texture	left	0.048	6.082	0.029	0.651	1.000	0.047

## Data Availability

The raw data supporting the conclusions of this article will be made available by the authors on request.
