# Correction: Sage et al. Hybridization of Acoustic and Visual Features of Polish Sibilants Produced by Children for Computer Speech Diagnosis. *Sensors* 2024, *24*, 5360

**DOI:** 10.3390/s24248061

**Published:** 2024-12-18

**Authors:** Agata Sage, Zuzanna Miodońska, Michał Kręcichwost, Paweł Badura

**Affiliations:** Faculty of Biomedical Engineering, Silesian University of Technology, Roosevelta 40, 41-800 Zabrze, Poland; zuzanna.miodonska@polsl.pl (Z.M.); michal.krecichwost@polsl.pl (M.K.); pawel.badura@polsl.pl (P.B.)

In the original publication [1], the funder Foundation for Polish Science (FNP) was not included. The correct Funding section appears below:

**Funding:** This work was supported by the National Science Centre, Poland, research project No. 2018/30/E/ST7/00525: “Hybrid System for Acquisition and Processing of Multimodal Signal in the Analysis of Sigmatism in Children”; partially by the Polish Ministry of Science, Poland, statutory financial support No. 07/010/BK_24/1034; and partially by the Foundation for Polish Science (FNP).

The authors state that the scientific conclusions are unaffected. This correction was approved by the Academic Editor. The original publication has also been updated.
